# A Gaussian-Distributed Quantum Random Number Generator Using Vacuum Shot Noise

**DOI:** 10.3390/e22060618

**Published:** 2020-06-02

**Authors:** Min Huang, Ziyang Chen, Yichen Zhang, Hong Guo

**Affiliations:** 1 Department of Electronics, and Center for Quantum Information Technology, State Key Laboratory of Advanced Optical Communication Systems and Networks, Peking University, Beijing 100871, China; iqehuangmin@pku.edu.cn (M.H.); chenziyang@pku.edu.cn (Z.C.); 2 State Key Laboratory of Information Photonics and Optical Communications, Beijing University of Posts and Telecommunications, Beijing 100876, China; zhangyc@bupt.edu.cn

**Keywords:** quantum random number generator, vacuum fluctuation, Gaussian distribution, goodness of fit test

## Abstract

Among all the methods of extracting randomness, quantum random number generators are promising for their genuine randomness. However, existing quantum random number generator schemes aim at generating sequences with a uniform distribution, which may not meet the requirements of specific applications such as a continuous-variable quantum key distribution system. In this paper, we demonstrate a practical quantum random number generation scheme directly generating Gaussian distributed random sequences based on measuring vacuum shot noise. Particularly, the impact of the sampling device in the practical system is analyzed. Furthermore, a related post-processing method, which maintains the fine distribution and autocorrelation properties of raw data, is exploited to extend the precision of generated Gaussian distributed random numbers to over 20 bits, making the sequences possible to be utilized by the following system with requiring high precision numbers. Finally, the results of normality and randomness tests prove that the generated sequences satisfy Gaussian distribution and can pass the randomness testing well.

## 1. Introduction

Random numbers are of extreme importance for a great range of applications from scientific to engineering fields, including statistical sampling, numerical simulation, lottery and cryptography. A typical example is the quantum key distribution (QKD), in which true random numbers are essential to guarantee its unconditional security [1,2,3,4]. The algorithm-based classical pseudo-random number generators have been widely applied for their simple implementation and extremely high generation rate [5]. However, the inherent determinacy of pseudo-random number generator makes it substantially deterministic and predictable, which leads to the failure in satisfying theoretically requirements of secure communication systems. Aside from the algorithmic method, extracting randomness from objective physical processes is feasible. An outstanding alternative is a quantum random number generator (QRNG), which exploits the intrinsic random nature of quantum mechanics [6,7], acts as a promising method in generating truly random numbers.

Practical (conventional) QRNG schemes could generate relatively large amount of quantum random numbers with high generation rate via utilizing easily accessible commercial devices. Schemes of various quantum random sources have been demonstrated, including discrete ones by measuring photon path [8,9,10], photon arrival time [11,12,13,14,15,16], photon number distribution [17,18], as well as continuous types of phase noise of lasers [19,20,21,22,23,24,25] and Raman scattering [21], intensity fluctuation of amplified spontaneous noise (ASE) [26,27,28,29], quadrature fluctuation of vacuum shot noise (VSN) [30,31,32,33,34], and other potential quantum state [35,36].

Practical schemes are proposed under assumptions only if the QRNG model works well, indicating the system is fully trusted. This rigorous condition can hardly be fulfilled, and device-independent (DI) protocols are proposed for closing the loophole. DI QRNG verify the randomness physically, taking the violation of Bell’s inequality [37,38] as a judgment [39,40]. Later, two branches are researched for alternative proposes, namely randomness extraction [41,42,43] and randomness amplification [44,45]. While DI protocols sacrifice too much on feasibility, a third choice which compromises between practical scheme and DI protocol is proposed. These semi-DI protocols merely make a reasonable assumption on critical devices [46,47,48,49,50,51], pursuing practical security instead of unconditional security.

The application fields of Gaussian RNG are diverse, in which the most significant application is a simulation, ranging from Monte Carlo method to simulation of communication channels and noises, biology, psychology, and so on. Specific to quantum information, Gaussian RNG provides Gaussian distributed random numbers for the modulation of coherent states in continuous-variable QKD systems [4,52,53]. However, all the previous QRNG schemes provide uniformly distributed random numbers. Despite the universality that uniformly distributed random numbers could be converted to any distributions mathematically, the conversion process itself somehow costs much time and resources. An even higher potential risk is the process is that approximate in principle [54], which may lead to the defects of performance in applications. In fact, most of the continuous-variable quantum random sources, owing to the central limit theorem, feature Gaussian distributed signals in the time domain, including vacuum shot noise and phase noise of laser. Hence, it is possible for hardware-based schemes, naturally including QRNG, to utilize the Gaussian distribution profile and directly generate random numbers as required.

In this paper, a practical scheme directly generating Gaussian distributed quantum random numbers is proposed. Here “directly” means there are no conversion steps from the uniform distribution to the Gaussian distribution, however, the scheme is not post-processing free. Firstly, we point out the inherent difference in entropy estimation for Gaussian distribution versus uniform distribution. Practical issues of sampling devices are discussed for entropy estimation and system optimization. Secondly, a novel post-processing method is proposed, which takes a step further from the recursive method in classical Gaussian distributed RNG [55]. It is designed to remove the impacts of classical noise in the system, along with fulfilling the precision and auto-correlation requirements from applications. Finally, an experimental setup is demonstrated to show the feasibility of this scheme, using vacuum fluctuation of the quantum state as a quantum random source, and the implementation has passed tests both on normality and randomness.

The structure of this article is described as follows. In Section 2, firstly we discuss the difference in entropy estimation between Gaussian and uniform distribution, followed by the analysis on the impacts of practical sampling device to the system, namely sampling range and sampling resolution. In Section 3, a novel post-processing method is proposed to overcome the disadvantage of low precision in sampling, and substantially eliminate the impacts of electronic noise. In Section 4, an experimental setup is demonstrated, as well as the optimization and post-processing operation on a practical system. Finally, the test results for both normality and randomness are shown.

## 2. Analysis of Gaussian Distribution QRNG Scheme

### 2.1. Gaussian Random Source and Entropy Estimation

#### 2.1.1. Vacuum Fluctuation

In principle, most quantum random sources with Gaussian distributed signals in the time domain can be applied in our scheme. Particularly, for the following excellent features, we choose vacuum fluctuation of the quantum state as the random source. Firstly, vacuum shot noise is caused by vacuum fluctuation, thus the randomness of the pure state is secured. Secondly, it is a Gaussian state, which means the measurement of either position or momentum quadrature $\hat{x}$ or $\hat{p}$ in a pure state will always follow a Gaussian distribution. Finally, it is identical, which means additional vacuum fluctuation introduced by devices, such as the beam splitter, will not affect the randomness of the quantum source.

The Wigner function of vacuum fluctuation is as follows:

(1) $$W_{0}\left(x,p\right)=\frac{1}{\pi }\exp\left(-x^{2}-p^{2}\right).$$

As a quasi-probability function, one can repeatedly measure either $\hat{x}$ or $\hat{p}$ quadrature, given fixed phase difference θ between the vacuum and LO signals. Taken $\hat{x}$ quadrature as an example, the probability density distribution (PDF) of detected signal should be:

(2) $${\left|\psi_{0}\left(x\right)\right|}^{2}=\int_{-\infty }^{+\infty }W_{0}\left(x,p\right)dp=\frac{1}{\sqrt{\pi }}\exp\left(-x^{2}\right),$$

which is perfectly Gaussian distributed, with mean value μ=0 and variance $\sigma^{2}=1/2$ centering at the origin in phase space.

Security is always an important issue to a cryptographic system, including quantum random number generator, compared to its classical counterpart. While there definitely exist some risks of leaking information to an adversary in randomness extraction, modeling of vacuum fluctuation also takes advantage of its property, of which it could never be tampered even by the most powerful adversary under the limitation of physical laws. Hence, unlike traditional applications of classical RNG, where the noises are usually treated as introduced by the system itself, we could regard any noise in the system as introduced by the eavesdropper (Eve) in the QRNG system, in attempt to reach a lower bound in entropy estimation.

For homodyne detection, signals of two balanced arms are subtracted to supress the common mode noise, while the amplification factor is decided by the system:

(3) $$\begin{array}{ccc} \Delta n & = & {\hat{n}}_{2}-{\hat{n}}_{1}=\hat{a}{\hat{a}}_{LO}^{†}+{\hat{a}}_{LO}{\hat{a}}^{†}\\ V_{samp} & \propto & A\Delta n \end{array}$$

(4) $$\begin{array}{cc} = & A\left|\alpha_{LO}\right|\left(\hat{x}\cos\theta +\hat{p}\sin\theta \right) \end{array}$$

where $\hat{a},{\hat{a}}^{†}$ are annihilation and creation operators, and $\hat{n}=\hat{a}{\hat{a}}^{†}$ is photon number operator. $V_{samp}$ is the signal at sampling device (after subtraction), *A* is the amplification factor of the system excluding LO signal, $\left|\alpha_{LO}\right|$ indicates the *X* quadrature of LO signal, and θ is the phase difference between vacuum and LO signal.

#### 2.1.2. Entropy Estimation

As a conventional scheme, entropy estimation should be done before randomness extraction. The most significant difference between uniform and Gaussian distribution, from the perspective of information theory, is that the information entropy H(X) should have different maximal value under different constraints.

In order to eavesdrop most information under classical scenario, Eve’s best strategy is figuring out $\max_{x\in X}p_{i}$, the highest probability of a single bin in a random variable *X*, which directly related to the minimal entropy (min-entropy):

(5) $$H\left(X\right)=-\log_{d}\max_{x\in X}p_{i}$$

where *d* is the base of logarithmic function that defines whether the signal is binary, decimal and so on. For binary information, we often define d=2. However, if the signal precision *n* is more than one bit, it could be also treated as $d=2^{n}$.

According to Equation (5), uniform distribution possesses the highest min-entropy with no constraints. Noticing that for the continuous case, the classical entropy H(X) always goes to infinity for ideal sampling device with infinite sampling range and precision. Therefore, we assume the total amount of information a single signal carries is 1, thus normalize the maximal value of information entropy rate. This method is also adopted for Gaussian distribution entropy estimation in the following analysis.

Meanwhile, in applications under certain constraints, namely the quadratic quantity of energy (or power) of signal is fixed, one is expecting a different PDF. This conclusion is naturally derived from the property that among all distributions with the same variance $\sigma_{m}^{2}$, Gaussian distribution possess the highest information entropy H(X):

(6) $$H\left(X\right)=\int_{-\infty }^{+\infty }-p\left(x\right)\log_{d}p\left(x\right)dx,$$

where p(x) is PDF of ideal Gaussian distribution.

This property indicates that, if the variance of continuous noise signal $\sigma_{m}^{2}$ is observable and steady, Gaussian distribution, instead of uniform distribution, could achieve a higher entropy. Fortunately, the variance of total noise is indeed measurable in a QRNG scheme, and perfectly matches the assumption. Therefore when adopting a Gaussian distributed random source, and the output is supposed to be Gaussian distributed, the information quantity acquired is significantly reduced during the conversion phase of uniform distributed RNG schemes.

To achieve a Gaussian distributed QRNG scheme, we should adopt the goodness of fit (GoF) test essentially, to verify whether the PDF of our samples are sufficiently close to Gaussian distribution. For Gaussian distribution, there are several specific methods, namely Kolmogorov-Smirnov test and Anderson-Darling test, which will be introduced detailed in Appendix A.

### 2.2. Impact of Sampling Device

In the entropy estimation phase, a similar idea of “worst-case scenario” to its uniform counterpart could be adopted. Alice loses some entropy due to the sampling device, while Eve may acquire original information from ideal Gaussian distribution. As a continuous distribution, either infinite sampling range or precision is not practical, and will cause the entropy to be infinite, hence we should set conditions considering the performance of the practical device.

Classical Gaussian RNG often set ±10σ as the bounds in high multiple-sigma test [56], it seems reasonable to follow this assumption. Meanwhile, sampling precision can hardly exceed 20 bits for current commercial analog-to-digital converter (ADC). Practical issues of range, precision and depth will be discussed in detail.

Despite the classical noise, our scheme is still a trusted device scheme, where the extractable randomness of the scheme is described as:

(7) $$R_{dis}=I\left(A:B\right),$$

where $R_{dis}$ refers to the generation rate of a QRNG, with “dis” indicates the discretized samples which may lose some information, and I(A:B) is the mutual information between the authorized users (Alice, Bob) in a cryptographic system. In QRNG scheme, specifically speaking, Alice is the random source and Bob is the randomness extractor. Apparently, QRNG could be (and in most occasions is) local, while Alice plays both roles of the sender (random source) and receiver (randomness extractor), thus I(A:B) is actually determined by the entropy H(A) of measured classical data.

Particularly, in the vacuum fluctuation scheme we demonstrated below, the variance of total noise $\sigma_{m}^{2}$ and the variance of classical noise $\sigma_{c}^{2}$ can be observed by separately turn on/off the LO signal. Since we believe the quantum noise *Q* and classical noise *E* are independent from each other, the min-entropy of quantum noise is a conditional entropy, with classical noise part *E* is given by [33]:

(8) $$\begin{array}{cc} H_{min}\left(M|E\right) & =-\log_{2}\left[\max_{e\in E}\max_{m\in M}P_{M|E}\left(m|e\right)\right]\\ & =-\log_{2}\max\left(c_{1},c_{2}\right) \end{array}$$

where M,E are the random variable of total measured noise and electric noise, m,e mean the specific measured value. R,δ means the sampling range and sampling resolution respectively. $c_{1}=\frac{1}{2}\left[\mathrm{erf}\left(\frac{e_{max}-R+3\delta /2}{2\sigma_{q}}\right)+1\right],c_{2}=\mathrm{erf}\left(\frac{\delta }{2\sqrt{2}\sigma_{q}}\right)$ refer to the two possible values that could be the maximal $p_{i}$ in Equation (5), and $e_{max}$ is the maximal possible electric noise.

Things differ a little under the Gaussian scenario comparing to the analysis in Ref. [33]. In the uniform scenario, the optimization for the system is setting $c_{1}=c_{2}$ to achieve maximal value of min-entropy. However, if we adopt $c_{1}=c_{2}$ in the Gaussian scheme, the raw data will definitely fail the GoF test. Therefore, we have to analyze the impact of the sampling device under the restriction of the GoF test, where there always exists $c_{1}<c_{2}$.

#### 2.2.1. Sampling Range

The sampling device will change the instantaneous voltages beneath (above) the lower (upper) threshold into $V_{min}$ (or $V_{max}$). Parameter *k* is the ratio between sampling range *R* and the deviation of signal σ. Finite sampling range will truncate probability distribution $P(x\geq \left|k\sigma \right|)$ outside the range ±kσ, another consequence is a significant defect at the tails, causing the PDF non-Gaussian.

We define a parameter called normalized min-entropy in our analysis. Supposing an ideal Gaussian distributed random variable, and the information carried by the variable is described as $H_{ideal-min}$ before normalized to 1. When taking practical sampling device into consideration, the distribution is changed and entropy is estimated by Equation (8), however, it should be monitored by the GoF test. In the following analysis of sampling range and resolution, the utmost assumption is that the signal should satisfy normality, meanwhile, this is also the assumption of the post-processing method below. Therefore, the min-entropy of distribution from a practical system should initially pass the GoF test, before it can be normalized according to the ideal case, which could be described as:

(9) $$H_{norm-min}=H_{min}\left(M|E\right)/H_{ideal-min}$$

**Figure 1:**
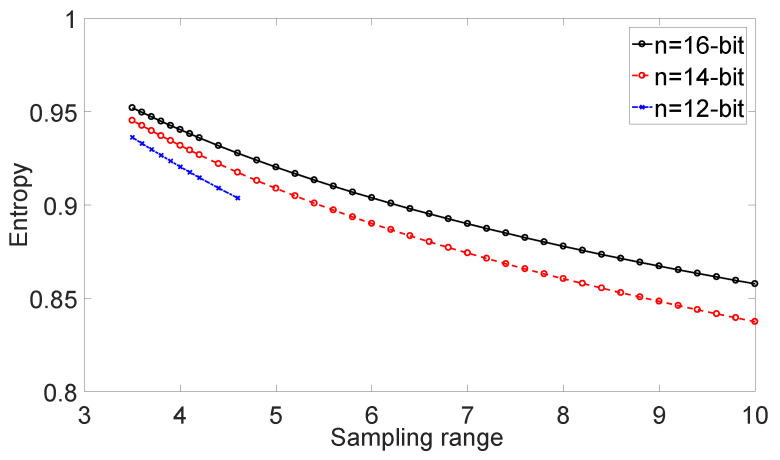
Sampling range versus normalized min-entropy. The figure draws a set of entropy curves calculated under the condition of sampling precision n=12,14,16 and sampled data size $L=10^{7}$ without noise. The observable value is the variance of total noise $\sigma_{m}^{2}$. All data should pass GoF test in prior.

Figure 1 shows the relationship between sampling range and entropy $H_{norm-min}$. Cases R≤±3.5σ are discarded for all precisions, due to these cases feature defected PDF and frequently fail the GoF test (with default significance level at α=0.01). However, lower sampling precision *n* with too large a sampling range will also fail the GoF test (as the curve n=12 stops at R=±4.6σ), since the discretization effect is notably increased for lower precision cases.

1. If *k* is too small, $V_{min}$ ($V_{max}$) will occur too often, making the random variable more predictable, and reducing entropy $H_{dis}\left(X\right)$. Furthermore, the worse profile of Gaussian distribution has a higher possibility to fail the GoF test, which does not match our requirement in post-processing and applications;
2. If *k* is too large, most signals will locate in a small range of sample bins, making the most significant bits (MSB) of samples more predictable, and also reducing entropy $H_{dis}\left(X\right)$. On the other hand, many sampling bins are unoccupied, wasting the ability of devices and substantially reduce the sampling precision.

For cases that successfully pass the GoF test, the normalized min-entropy decreases as the sampling range increases. The curves do have a period of rapid increase under no constraint assumption within the range of R≤±3σ, however, these cases are rejected by the GoF test. From the view of variance, as long as the raw data pass GoF test, the higher *k* value is, the lower normalized min-entropy is, which shows great significance on the matching of signals and range of sampling device.

#### 2.2.2. Sampling Resolution

Finite sampling resolution δ will result in information loss of probability distribution inside the minimal discrete sampling interval, i.e., resolution: $\left[x_{i}-1/2\delta ,x_{i}+1/2\delta \right]\left(i\in \left[0,2^{n}-1\right]\right)$. Intuitively, entropy grows monotonously as the precision *n* increases. If *n* is too small, too many detailed information is lost, and we can hardly extract random numbers after entropy estimation.

**Figure 2:**
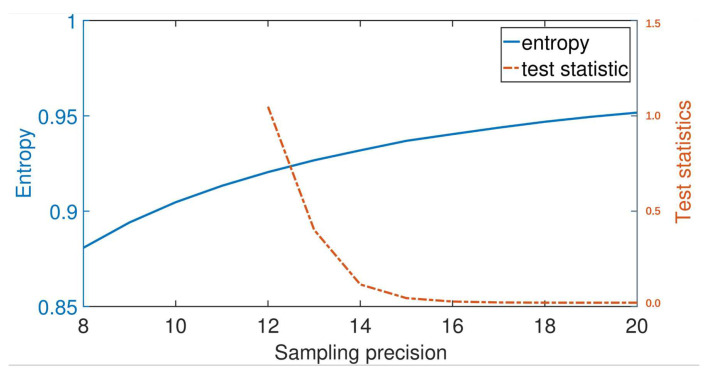
Sampling precision versus normalized min-entropy. The figure is calculated under conditions of sampling range R=±4σ and sampled data size $L=10^{7}$ without noise. The observable value is the variance of total noise $\sigma_{m}^{2}$. All data should pass goodness of fit (GoF) test in prior.

Figure 2 shows the relationship between precision and entropy $H_{norm-min}$. For the same reason discussed in sampling range analysis, cases R≤±3.5σ are discarded. Despite precision below n=12 will frequently fail the GoF test due to a strong discretization effect, we estimate the entropy to show the trend of entropy curve.

#### 2.2.3. Sampling Depth

Sampling depth (maximal samples in a single buffer) mainly affects the practical system on the GoF test. As the test statistic shows, the AD test is distribution-free, but sample data size *L* related. An identical distribution with sample length 10 times larger would lead to approximately linearly increased test statistic, while the critical value remains the same. This is due to it possessing a larger sample space, any violation on the PDF becomes more significant to be detected by the GoF test. Therefore, a larger buffer should have a better PDF for raw data to pass the GoF test.

According to the three factors discussed above, we consider R=±4σ to be the optimal sampling range for noise-free cases, while the precision and depth should be as high as possible, which is not so crucial in uniform occasions. It is highly recommended that if one wants to achieve a Gaussian distribution QRNG, sampling precision should be at least 12 bits for decent performances. Although the sampling range of the practical device is often fixed, one can adjust the amplification factor $A\left|\alpha_{LO}\right|$ to adapt the range, aiming at achieving better performance. However, noise introduced by the system with a variance of $\sigma_{c}^{2}$ often alters the PDF and optimization condition. If the noise introduced by the system is not crucial enough to change the PDF, the following post-processing method could significantly reduce its influence.

## 3. Post-Processing

Post-processing is an essential part in QRNG scheme. It is adopted to remove the impacts of classical noise in the system as well as the imperfections caused by finite sampling. Most of the post-processing methods can also improve the probability distribution of the raw data.

The Toeplitz matrix hashing method [23,57,58] is widely acknowledged as the most effective method in QRNG post-processing. However, the whole method aims at uniform distribution generation [59], hence does not meet our requirement. Here we propose another post-processing method originating from recursive method [55] adopted in classical Gaussian distribution RNG schemes.

The recursive method takes the essence of Gaussian distribution that is, the summation of any amount of Gaussian distributed variables is still Gaussian distributed:

(10) $$Y=\sum_{i}k_{i}X_{i},$$

while the original Gaussian variables $X_{i}$ satisfy $X_{i}=N\left(\mu_{i},\sigma_{i}^{2}\right)$, the output *Y* should satisfy $Y=N(\sum_{i}k_{i}\mu_{i},\sum_{i}k_{i}^{2}\sigma_{i}^{2})$.

Traditional central limit theorem (CLT) of non-Gaussian cases is only valid for a large amount of independent identical distributed (i.i.d.) variables. On the contrary, we notice that the recursive method takes merely four elements as Equation (11) shows. By adopting the recursive method, one could avoid the risk that raw data of distinguishable non-Gaussian variables are converted to identical Gaussian distribution.

The original transfer matrix $T_{\mathrm{rec}}$ is derived from following operations:

(11) $$\begin{array}{cc} a_{1}^{\prime } & =c-a_{3},\\ a_{2}^{\prime } & =c-a_{2},\\ a_{3}^{\prime } & =a_{1}-c,\\ a_{4}^{\prime } & =a_{4}-c, \end{array}$$

where $c=\frac{1}{2}\sum_{i=1}^{4}a_{i}$. Thus we can denote the relationship between input and output vectors $A_{i},A_{i}^{\prime }$, as well as the operating matrix $T_{rec}$ ($\frac{1}{2}$ is normalization coefficient):

(12) $$\begin{array}{ccc} A_{i}^{\prime } & = & T_{\mathrm{rec}}A_{i}, \end{array}$$

(13) $$\begin{array}{ccc} T_{rec} & = & \frac{1}{2}\left[\begin{array}{cccc} 1 & 1 & -1 & 1\\ 1 & -1 & 1 & 1\\ 1 & -1 & -1 & -1\\ -1 & -1 & -1 & 1 \end{array}\right] \end{array}$$

The output of the recursive method possesses the perfect auto-correlation property. However, it cannot extend the precision of a single number. To make full utilization of all significant bits from different raw data in precision extension, adding random numbers from i.i.d. Gaussian distributed variables with different weight is an effective method.

The post-processing method includes two steps. Firstly, we utilize the *m*-MSB (most significant method) as pre-processing. When entropy estimation phase introduced in Section 2 is done, the value *m* utilized in *m*-MSB processing is:

(14) $$m=\lfloor H_{min}\left(M|E\right)\rfloor .$$

Then we should adopt an operation that could achieve precision extension based on the matrix in Equation (13). Noticing that since the raw data has passed GoF test, the condition in Equation (10) is satisfied.

Assuming *X* is the original variable from ADC, and we could divide *X* into groups of Gaussian distributed variables $X_{i}$, before taking operation as Equation (10) shows. As an example where we divide the raw data into l=4 groups, consecutive four random numbers $x_{4i-3},x_{4i-2},x_{4i-1},x_{4i}$ will form a vector $A_{i}$ before operating by the matrix. Particularly in Equation (10), suppose $k_{i}=2^{-i}$, then every adjacent raw data in vector $A_{i}$ shifts only 1 more bit, thus the summation has a precision of n=m+l−1 bit, while m,n are the precision of variables $X_{i}$ and *Y* respectively, and *l* is the number of groups.

Combining the analyses above, we modify $T_{rec}$, adding different weights in the matrix similar to the original method:

(15) $$\begin{array}{cc} a_{1}^{\prime \prime } & =1/2a_{1}+1/4a_{2}-1/8a_{3}+1/16a_{4},\\ a_{2}^{\prime \prime } & =1/16a_{1}-1/2a_{2}+1/4a_{3}+1/8a_{4},\\ a_{3}^{\prime \prime } & =1/8a_{1}-1/16a_{2}-1/2a_{3}-1/4a_{4},\\ a_{4}^{\prime \prime } & =-1/4a_{1}-1/8a_{2}-1/16a_{3}+1/2a_{4}, \end{array}$$

Thus we can denote ($k_{\mathrm{NC}}$ is normalized coefficient):

(16) $$\begin{array}{ccc} A_{i}^{\prime \prime } & = & S_{rec}A_{i}, \end{array}$$

(17) $$\begin{array}{ccc} S_{rec} & = & k_{\mathrm{NC}}\left[\begin{array}{cccc} 1/2 & 1/4 & -1/8 & 1/16\\ 1/16 & -1/2 & 1/4 & 1/8\\ 1/8 & -1/16 & -1/2 & -1/4\\ -1/4 & -1/8 & -1/16 & 1/2 \end{array}\right] \end{array}$$

Noticing that, the structure of $S_{rec}$ is very much similar to the original structure of $T_{rec}$. Both of them share two rows/columns with three positive and one negative element, and others with three negative and one positive. This type of structure is convenient for expansion to a 8×8 or even larger size of $T_{rec}$ [55]. For $S_{rec}$, the expansion method is similar, as long as obeying the rules discussed below.

A crucial difference between the original recursive method and our modified method is that, since we introduce different (absolute) value in the operating matrix, the auto-correlation coefficient will not remain flat. Therefore, we can only extract one number from $A_{i}^{\prime \prime }$ (of n=m+l−1 bit precision, where *m* is the precision of $A_{i}$, *n* is the precision of $A_{i}^{\prime \prime }$ as the final output, and *l* is the size of $S_{rec}$), while in the original case all numbers of $A_{i}^{\prime }$ (of *m* bit precision) could be extracted.

The recursive method post-processing operation can be designed, hence it is definitely more flexible than Equation (10). Utilizing matrix for a precision extension instead of simply adding i.i.d. Gaussian distributed variables have several merits:

1. Elements in the matrix, which are the weights in Equation (10), is not fixed, as long as they obey fundamental rules. For 4×4 matrix, each row/column should have 3 (1) positive and 1 (3) negative elements, and the position should not be the same; the absolute value of each row and column should not be the same either. Thus there is a group of $S_{rec}$ with hundreds of possible matrices;
2. The size of the matrix can be designed, which indicates how many raw numbers will be used to generate a final number. We take the 4×4 matrix as the simplest example for a demonstration. However, when the precision after *m*-MSB pre-processing is inadequate, and a larger matrix should be made. For instance, in the following section of implementation, we generate 12-bit Gaussian distribution numbers from 5-bit pre-processed data, by utilizing an 8×8 matrix. If the matrix size is larger, it has a potential for even higher precision, such as five-bit pre-processed data with a 16×16 matrix will generate 20-bit Gaussian distribution random numbers for high multiple-sigma applications.
3. The values of matrix elements can also be designed, which indicate shifted bits of the pre-processed data. In the discussion above, weights of adjacent numbers always follow the power of 1/2, which means that adjacent numbers in $A_{i}$ should shift one bit in the summation operation. However, if we change 1/2 to 1/4, it means that adjacent numbers in $A_{i}$ should shift two bits. Remember that according to Equation (17), a normalized coefficient $k_{\mathrm{NC}}$ should be carefully calculated to match the designation, making sure that the input and output share the same variance.

Due to these merits above, one can design his/her own $S_{rec}$ matrix for alternative experimental setup and application requirements. Furthermore, these properties leave huge space for further introduction of pre-generated random seed. It is possible to prepare several operating matrices and, based on the random seed that generated before or even feedback from real-time QRNG scheme, alter the post-processing operation in real-time.

Table 1 shows the relative entropy H(p(x)|q(x)) between p(x) and q(x). p(x) is quasi-Gaussian distributed, mixing ideal Gaussian distribution with several types of classical noise of small variance. q(x) is the reference of standard Gaussian distribution. It is clear that the post-processing method dramatically reduces the impact of noise for low Quantum-to-Classical Noise Ratio (QCNR) cases, especially for those noises which a not Gaussian, regardless of the profile of raw data. However, noise is still distinguishable from a standard Gaussian distribution.

## 4. Implementation and Results

### 4.1. Experimental Setup

**Figure 3:**
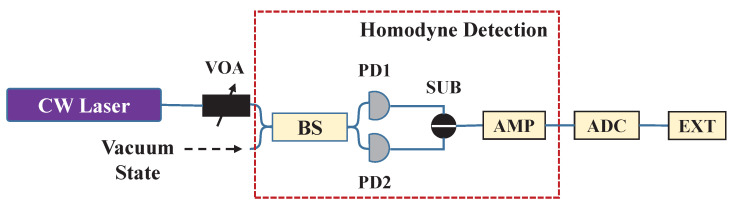
The schematic setup of vacuum shot noise based Gaussian distribution QRNG. Vacuum State: Vacuum shot noise (as random source); CW Laser: Continuous wave Laser (as local oscillator); VOA: Variable Optical Attenuator; BS: 50:50 Beam Splitter; PD1, PD2: Photodiode detectors (as balanced detector in homodyne detection); SUB: Subtractor; AMP: Amplifier; ADC: Analog-to-Digital Converter; EXT: Randomness Extractor.

**Figure 4:**
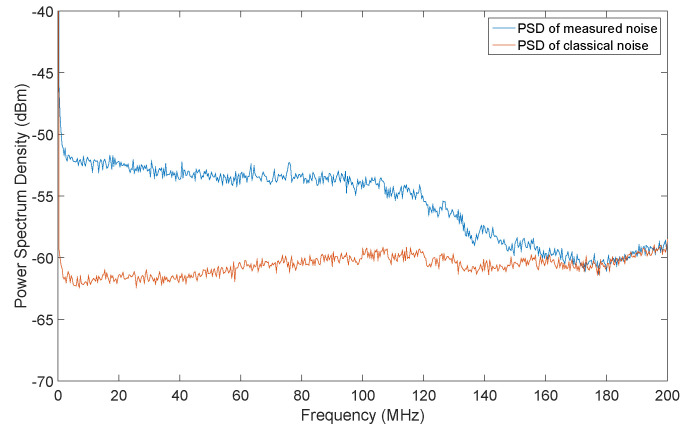
The power spectral density (PSD) function of the vacuum fluctuation when the LO is on (blue line) and off (red line). Mean value of total measured noise is −52 dB, while mean value of classical noise is −61 dB. The quantum noise dominates by over 10 dB with AC coupling, and possesses a flat spectrum within system frequency limited by the homodyne detectors (1 kHz–100 MHz). The 3 dB bandwidth of detector is 100 MHz.

We experimentally demonstrate our scheme and the setup is described as follows (as shown in Figure 3). The local oscillator (LO) is 1550 nm distributed feedback laser (NKT Basic E15, linewidth 100 Hz) with adjustable output power up to 15 mW, connecting to an external variable optical attenuator (VOA) precisely setting the amplification factor of the LO signal. Vacuum shot noise, physically provided by blocking one input port of a 50:50 beam splitter (BS), interferes with the LO light. The signals are sent to a well-tuned homemade AC coupling homodyne detector (measurement bandwidth limited to 100 MHz by low-pass filter) to measure the noise. Following circuits including an analog-to-digital converter (ADC, ADS5400, sampling frequency 200 MHz, sampling precision 12 bits and input voltage range 1.5 V peak-to-peak), a field-programmable gate array (FPGA, KC705 evaluation board) that realizes randomness extraction and data precision adjustment. The power spectral density function of total noise and classical noise is shown in Figure 4.

To obtain better performance, the power of LO light was examined by setting different LO power with fixed steps. When LO light was off, the vacuum fluctuation can be ignored, and classical noise contributes to the output with variance of $\sigma_{c}^{2}$, which is quite steady. The variance of total noise $\sigma_{m}^{2}$ increases as LO power (after VOA) getting stronger. The linear region ends when LO power increases at around 9.5 mW, and finally saturates at around 13 mW.

Our system features a high QCNR to obtain more potential information from the signal. By setting LO power slightly less than saturation at around 12 mW (6 mW for each branch of the balanced detector), we have acquired 12-bit raw data after ADC, and calculate the variance of signals when LO light is on/off, representing the total and classical noise respectively. Noticing that, all the units mentioned here are sampling bins, and according to the ability of our sampling device, one sampling bin roughly equals 0.366 mV.

Firstly, due to the fixed sampling range, $V_{\mathrm{range}}=2^{12}$ and peak-to-peak value is $V_{p-p}=200$, around 3-bit MSB is discarded. Since the variance of total noise is $\sigma_{m}^{2}=1200.7$, classical noise is $\sigma_{c}^{2}=82.5$, variance of quantum noise can be calculated: $\sigma_{q}^{2}=1118.2$, thus the maximal QCNR is defined by:

(18) $$\gamma =\mathrm{QCNR}=\frac{\sigma_{m}^{2}-\sigma_{c}^{2}}{\sigma_{c}^{2}}=13.55,$$

with QCNR=13.55(11.3dB), the classical noise after normalization is ε=1/QCNR=0.074.

As QCNR indicates, classical noise can only fluctuate in a small range of voltage. The MSB part of the residual sample is more likely to be affected by quantum noise, while the LSB part is affected by both quantum and classical noise, which is opposite to uniform distributed occasions.

We adopted entropy estimation initially in the post-processing phase. Our ADC has a sampling range of 1.5 V peak-to-peak and sampling precision of 12 bits, thus the quantization error is ${\left(\delta /12\right)}^{2}=9.3132\times 10^{-10}V^{2}$. While the LO is turned off, measured voltage variance is $\sigma_{c}^{2}=1.11\times 10^{-5}V^{2}=82.5\delta^{2}$, and the total measured voltage variance is $\sigma_{m}^{2}=1.61\times 10^{-4}V^{2}=1200.7\delta^{2}$. Since the requirement of passing GoF test, the system always works under safety condition $c_{1}<c_{2}$ in Equation (8), hence the min-entropy is determined by the middle of the distribution, i.e., $H_{min}=-\log_{2}\left(\mathrm{erf}\left(\delta /2\sqrt{2}\sigma_{q}\right)\right)=6.39$ bits. Therefore, the rest 12-3-6 = 3-bit LSB is doubtful for security aspect, and its influence should be eliminated by post-processing. To make our scheme more reserved, we keep five bits per signal from the highest non-zero MSB as the pre-processed data in precision extension. Hence, the output has a precision of 5 + (8 −1) = 12 bit per signal, while the generation rate by number is 1/8 of the original sampling rate, i.e., 25 M samples per second.

We compare our scheme in generation rate with traditional method of uniform distribution QRNG plus inverse CDF conversion post-processing. Under the condition of same implementation settings, namely sampling rate $f_{s}$, sampling precision *n* and min-entropy (extractable quantity of randomness) H(x), traditional method can generate $f_{s}n$ raw data, and around $f_{s}n\cdot H\left(x\right)$ final data in uniform distribution with estimated entropy H(x), thus the generation rate of *k*-bit Gaussian distributed number is $f_{s}n\cdot H\left(x\right)/k$. On the other hand, our scheme provide $f_{s}$ raw data, and around $f_{s}/4$ or $f_{s}/8$ final data of Gaussian distributed numbers. Considering the practical condition n=12, H(x)=0.6∼0.8 and k=12∼32, n·H(x)/k and 1/4 are approximately at the same order of magnitude. Hence, the generation rate of two schemes are leveled, but our scheme has avoided the enormous time cost to calculate the accurate Gaussian distributed value, or space cost to store the huge library of inverse CDF conversion in post-processing [55].

### 4.2. Test Results

#### Normality Tests

**Figure 5:**
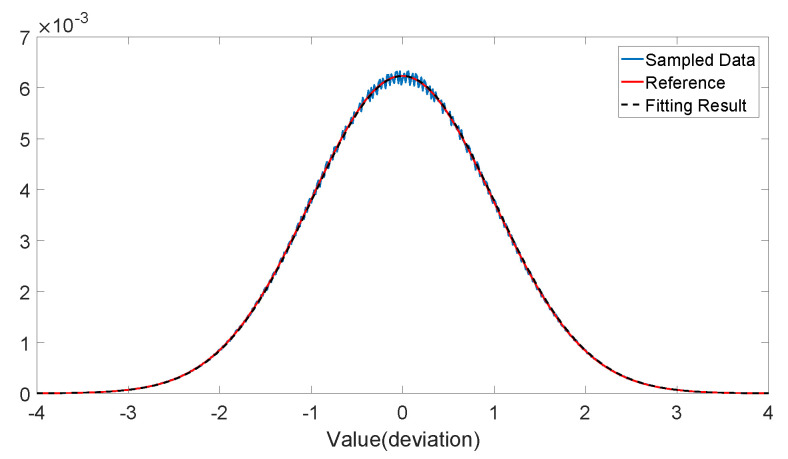
Result of distribution fitting for the random sequences after post-processing. Blue and red solid lines refer to sampled data and reference (ideal vacuum noise with certain variance $\sigma_{q}^{2}$) calculated above, and dashed line refers to fitting result. The *R*-square parameter of fitting is R=0.9997. Reference and fitting curves are nearly indistinguishable.

Initially, the random sequences after post-processing should pass the normality test. The fitting result is shown in Figure 5. Random sequences also pass several goodness of fit tests, the test result is shown in Table 2.

We calculate the 3σ threshold of bias e(n) and auto-correlation $a_{k}\left(n\right)$ under Gaussian distribution. 3σ criterion is a rough threshold indicating the bias e(n) and auto-correlation coefficient $a_{k}\left(n\right)$ of a finite sample from ideal random sequence, should only exceed the reference by a probability of $1-\mathrm{erf}\left(\frac{3}{\sqrt{2}}\right)=0.3\%$.

The 3σ criterion originates from the central limit theorem (CLT). The traditional description of CLT indicates that, the summation $S_{n}$ of a large amount of i.i.d. variables $\left\{X_{i}\right\}$ should always have asymtotic behavior to Gaussian distribution:

(19) $$Z_{n}=\frac{S_{n}-E\left(S_{n}\right)}{\sqrt{D(S_{n})}}\to N\left(0,1\right),$$

where $S_{n}=\frac{1}{n}\sum_{i}X_{i}$.

As long as we can derive the mean value μ and variance $\sigma^{2}$ of certain test statistic, the 3σ threshold is determined. These two statistics can be described as:

(20) $$\begin{array}{cc} e(n) & =\frac{1}{N}\sum_{i=1}^{N}\left(s_{i}-\bar{s}\right),\\ a_{k}\left(n\right) & =\frac{\sum_{i=1}^{N}\left(s_{i}-\bar{s}\right)\left(s_{(i+k)\;\mathrm{mod}N}-\bar{s}\right)}{\sum_{i=1}^{N}{\left(s_{i}-\bar{s}\right)}^{2}}, \end{array}$$

while for the Gaussian distribution, there exists: $\bar{s}=\mu =0,\frac{1}{N}\sum_{i=1}^{N}{\left(s_{i}-\bar{s}\right)}^{2}=\sigma^{2}=1$. Hence the simplification of Equation (20) is:

(21) $$\begin{array}{cc} e(n) & =\frac{1}{N}\sum_{i=1}^{N}s_{i},\\ a_{k}\left(n\right) & =\frac{\sum_{i=1}^{N}s_{i}s_{(i+k)\;\mathrm{mod}N}}{\sum_{i=1}^{N}s_{i}^{2}}, \end{array}$$

**Figure 6:**
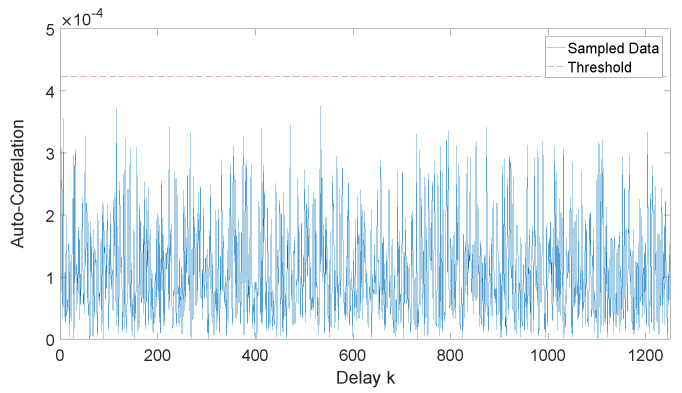
Result of 3σ test of auto-correlation $\left|a_{k}\left(n\right)\right|$ versus delay k≠0 for Gaussian distribution random sequence. Data size is 50M samples, data precision is 12 bit. Threshold is derived as $\left|a_{\mathrm{th}}\left(n\right)\right|=3/\sqrt{n}$ as above ($3/\sqrt{n}=4.2\times 10^{-4}$).

One can easily derive that for Gaussian distribution, bias follows distribution $e\left(n\right)∼N\left(\mu ,\frac{1}{n}\sigma^{2}\right)$, while auto-correlation follows distribution $a_{k}\left(n\right)∼N\left(0,\frac{1}{n}\right)$ (and free from *k*), both of which are normal distribution. We utilize the threshold of 3σ criterion to test our Gaussian distribution QRNG, and the result of auto-correlation coefficient is shown in Figure 6.

In addition, since no test suites for Gaussian distributed random numbers are proposed, we converted some random sequences into uniform distribution for randomness test. The conversion is done by CDF method discussed in Appendix B, and result is shown in Table 3.

## 5. Conclusions

We proposed a QRNG scheme generating random numbers with a Gaussian distribution based on vacuum fluctuation of a quantum state, a theoretically proved Gaussian distributed random source. We analyzed the impacts of practical issues in the QRNG system, including sampling range, resolution and depth of the sampling device, along with the optimization method. A novel and flexible post-processing method is proposed, inspired from the classical RNG scheme, to extend the precision of a single number to 12, or even over 20 bits, where the property of Gaussian distributed PDF and the auto-correlation coefficient is maintained at the cost of generation rate. The generated random sequence simultaneously pass normality test focusing on distribution, as well as widely acknowledged NIST-STS test suite of randomness (after converted to uniformly distributed sequences). We experimentally demonstrated the scheme based on vacuum shot noise with conventional devices at a generation rate 25M of sample per second.

Our scheme takes advantage of the Gaussian distributed profile of quantum random sources. Impacts of practical issues, strictly monitored by the GoF test, could not essentially alter the profile of Gaussian distribution, and consequently eliminated by the designed fast post-processing method. We provide a novel method generating Gaussian distribution random numbers effectively.

We have to admit that, despite other QRNG schemes would face consumption in uniform-Gaussian conversion procedure, the generation rate in our system is questionably inadequate for a practical continuous-variable QKD system [52,53]. However, we demonstrate the feasibility of such kind of QRNG, and two factors limiting the generation rate, both of which have huge space to improve. Firstly, the frequency in our system is quite low, due to the limitation of detector bandwidth in our system. By using a balanced detector and ADC with higher bandwidth, the generation rate can be further improved by at least one order of magnitude. Secondly, despite the amplification, the amplified vacuum fluctuation is still too small compared with the sampling range, thus fails to make full use of the ADC.

Security is another issue that is extremely significant to the QRNG system. Despite that, we have estimated the min-entropy in the trusted device scenario and operate accordingly, it is not totally clear whether the MSB method in the post-processing phase eliminates the classical noise substantially. The security issue of Gaussian-distributed schemes needs further discussion, and we are keen on tracing related works.

## Figures and Tables

**Table 1:** The relative entropy H(p(x)|q(x)) between unknown distribution p(x) (with normalized variance) and reference q(x) after post-processing. All data unit is $10^{-5}$. $H_{\mathrm{rel}}=0$ means the unknown distribution is identical with q(x). We assume p(x) a standard Gaussian distribution with minor classical noise of Quantum-to-Classical Noise Ratio (QCNR) ranges between 3–20 dB. In order to highlight the smooth effect on profile, data is designed for small size with $n_{\mathrm{tot}}=10M$. The residual relative entropy after post-processing is possibly due to the finite size effect of this calculation method.

	Normal		t-Dist.		Uniform		Rayleigh	
QCNR(dB)	Before	After	Before	After	Before	After	Before	After
3	1.2225	1.1653	1.2966	1.3338	64.036	4.6896	179.40	1.4993
6	1.2582	1.2320	1.4416	1.4348	9.0556	1.4507	39.991	1.2510
10	1.1920	1.2031	1.2478	1.2741	1.4064	1.3917	4.5185	1.1799
20	1.2717	1.2455	1.2132	1.2510	1.1764	1.1996	1.2150	1.1964

**Table 2:** The result of commonly used matlab functions for normality test. Random sequences are normalized to N(0,1) for the convenience of matlab tests, so there are no terms of variance test.

Function	Mean	AD Test	JB Test	*t*-Test
Calculated result	−$3.6066\times 10^{-4}$	*p* = 0.4788	*p* = 0.3678	*p* = 0.2023
Confidence Interval	[−0.0036, 0.0036]	NULL	NULL	NULL
Hypothesis value	H=0	H=0	H=0	H=0
Status	Pass	Pass	Pass	Pass

**Table 3:** The result of NIST-STS test after CDF conversion of Gaussian distributed random sequences. For the test environment of significance level α=0.01 and block number of n=400, The *p*-value should be over p=0.01 threshold (uniformity version over 0.0001) and proportion should be within the range [0.9750,1].

Test Name	*p*-Value	Proportion	Status
Frequency	0.811993	394	Success
Block Frequency	0.719747	396	Success
Cumulative Sums	0.785103(KS)	395.5(avg)	Success
Runs	0.270275	396	Success
Longest Run	0.788728	397	Success
Rank	0.375313	396	Success
FFT	0.272297	395	Success
Non-overlapping	0.647530(KS)	394(avg)	Success
Overlapping	0.830808	396	Success
Universal	0.451234	393	Success
Approx. Entropy	0.739918	397	Success
Excursions	0.726852(KS)	392(avg)	Success
Excursions Var.	0.670396(KS)	395(avg)	Success
Serial	0.589359(KS)	392.5(avg)	Success
Complexity	0.124115	392	Success

## Abbreviations

The following abbreviations are used in this manuscript:

QRNG	Quantum Random Number Generator
QKD	Quantum Key Distribution
PDF/CDF	Probability/Cumulative Density Function
ADC	Analog-to-Digital Converter
QCNR	Quantum-to-Classical Noise Ratio
GoF	Goodness of Fit
MSB/LSB	Most/Least Significant Bit

## Appendix A. Goodness of Fit Tests

Kolmogorov-Smirnov(KS) test is the primitive one-parameter GoF test for Gaussian distribution [60]. KS test describe the closeness by distance:

(A1) $$D_{i}=\max\left(F\left(Y_{i}\right)-\frac{i-1}{N},\frac{i}{N}-F\left(Y_{i}\right)\right),$$

where $Y_{i}$ is the sample data in ascending order, F(·) is the CDF, *N* is the size of sample. The final test statistic is the maximal distance among $D_{i}$: $D=\max D_{i}$, with significance level of α=0.01.

Unfortunately, Gaussian distributed QRNG should pay extremely high attention to the tails of PDF, due to large amount of information they bring, of which KS test can not indicate properly. Therefore, a modified version of GoF, the Anderson-Darling(AD) test is proposed [61]:

(A2) $$A^{2}=-N-\sum_{i=1}^{N}\frac{2i-1}{N}\left[\ln F\left(Y_{i}\right)+\ln\left(1-F\left(Y_{N+1-i}\right)\right)\right],$$

where $A^{2}$ is the test statistic.

AD test has a critical value that only relates to α and distribution-free. In practice, we choose the AD test over its alternatives (KS test as well as other GoF tests, namely Jarque–Bela, Lilliefors tests, etc.) as the main GoF test, for its convenience, universality and high sensitivity towards profile [62].

## Appendix B. PDF Conversion between Uniform and Gaussian Distribution

Several methods are proposed for uniform-Gaussian (or inverse) conversion. Two of them outstand for their clear expressions:

1. Box-Muller [63]: uniform and Gaussian distribution can be easily converted between rectangular basis and polar basis. Assuming that U,V are uniform variables, and X,Y are Gaussian variables, there exist:
  (A3) $$\begin{array}{cc} X & =\sqrt{-2\ln U}\sin\left(2\pi V\right),\\ Y & =\sqrt{-2\ln U}\cos\left(2\pi V\right), \end{array}$$
  while for the inverse conversion:
  (A4) $$\begin{array}{cc} U & =\exp(-\frac{X^{2}+Y^{2}}{2}),\\ V & =\frac{1}{2\pi }\arctan\frac{X}{Y}. \end{array}$$
2. CDF method [54]: uniform and Gaussian distribution can be converted by cumulative density function (CDF) and its inverse function, ICDF. Assuming *U* an uniform variable, and *X* a Gaussian variable, there exist:
  (A5) $$\begin{array}{c} \begin{array}{c} U=\mathrm{CDF}(X),\\ X=\mathrm{ICDF}(U), \end{array} \end{array}$$
  CDF(·) is denoted as:
  (A6) $$\mathrm{CDF}(X)=\int_{-\infty }^{x}p\left(x\right)dx=\frac{1}{2}\left[1+\mathrm{erf}(\frac{x}{\sqrt{2}})\right].$$

In the last part of our randomness test, we choose the CDF method for its convenience in numerical calculation. However, when utilizing the ICDF method as the ordinary Gaussian distribution QRNG scheme, it still takes a large number of resources, compared to our post-processing method.
